# Establishing tau-PET cut-points for cognitive diagnosis with ^18^ F-PI-2620 in a multi-ethnoracial cohort

**DOI:** 10.1162/IMAG.a.41

**Published:** 2025-06-16

**Authors:** Victoria R. Tennant, Koral V. Wheeler, Noelle N. Lee, Jamie A. Terner, Maxwell W. Hand, Suchita Ganesan, Patrick Walsh, Aisha Greene, Tyler Berkness, Tiantian Lei, Rema Raman, Robert A. Rissman, Bradley T. Christian, Melissa Petersen, Ann D. Cohen, Beau M. Ances, Karin L. Meeker, Zhengyang Zhou, Rajesh R. Nandy, Kristine Yaffe, Sid E. O’Bryant, Arthur W. Toga, Meredith N. Braskie

**Affiliations:** Mark and Mary Stevens Neuroimaging and Informatics Institute, Keck School of Medicine, University of Southern California, Los Angeles, CA, United States; Alzheimer’s Therapeutic Research Institute, University of Southern California, San Diego, CA, United States; Waisman Center, University of Wisconsin-Madison, Madison, WI, United States; University of North Texas Health Science Center at Fort Worth, Fort Worth, TX, United States; University of Pittsburgh School of Medicine, Pittsburgh, PA, United States; Washington University School of Medicine in St. Louis, St. Louis, MO, United States; Department of Psychiatry, Neurology, and Epidemiology, Biostatistics University of California, San Francisco, CA, United States

**Keywords:** tau, amyloid, PET, Alzheimer’s disease, aging

## Abstract

^18^F-PI-2620 is a newer tau-PET tracer with minimal off-target binding in thechoroid plexus. Defining tau cut-points to differentiate between cognitively unimpaired andimpaired individuals is crucial for both biomarker validation and clinical use, but researchusing^18^F-PI-2620 is limited. In 675 participants (mean age: 64, 64% Female, 186Hispanic, 209 non-Hispanic Black, and 280 non-Hispanic White) from the Health and Aging BrainStudy-Health Disparities, we used the area under the receiver operating characteristic curve toidentify a region of interest and corresponding cut-point at which^18^F-PI-2620standardized uptake value ratio best distinguished between amyloid negative cognitivelyunimpaired and amyloid-positive cognitively-impaired individuals. The regional or compositestandardized uptake value ratio that maximized sensitivity and specificity (measured by theYouden index) was selected as the best-performing region of interest and was further evaluatedusing Gaussian mixture modeling. We evaluated the performance of the chosen region of interestand cut-point in the three ethnoracial groups. The best-performing region of interest was themedial temporal composite, with a cut-point of 1.26. This region performed well in Hispanic andnon-Hispanic White subgroups, but not in the non-Hispanic Black subgroup. The data show theutility of this region to identify clinically relevant levels of tau. Future work shouldexplore the relationship of tau to comorbid conditions across ethnic and racial groups.

## 1 Introduction

The primary current biomarker model of Alzheimer’s disease (AD) proposes a sequential,non-linear series of pathological changes in which amyloid beta (Aβ) becomes abnormalfirst but does not produce clinical deficits, followed by the spread of tau out of the medialtemporal lobe to wider neocortical regions (Jack,Wiste, et al., 2018;Jack Jr. et al.,2024;Jagust, 2018;La Joie et al., 2020;Schöll et al., 2016), although the pattern and progression ofpathological changes may vary across different tau subtypes (Charil et al., 2019;Ferreiraet al., 2020;Murray et al., 2011). TheAβ, tau, and neurodegeneration (AT(N)) framework (Jack, Bennett, et al., 2018;Jack et al., 2024) systematizes these biological changes for research feasibility.Staging within this framework relies on determining cut-points for biomarker abnormality, butbecause these thresholds are influenced by the study sample, their applicability to more diversepopulations may be limited (Gleason et al.,2022).

Tau accumulation is more closely linked to cognitive changes than Aβ, with higherbaseline tau PET associated with steeper rates of cognitive decline over time (Hanseeuw et al., 2019;Jack et al., 2019;La Joie etal., 2020). Accordingly, establishing cut-points for tau positivity is critical fortracking disease progression, staging, and evaluating intervention success (Jack, Bennett, et al., 2018;Jack et al., 2017). Several ongoing studies arefocused on characterizing later-developed tau-PET tracers, including^18^F-PI-2620,^18^F-MK-6240, and^18^F-RO-948 (Betthauser et al., 2019;Kroth et al.,2019;Mormino et al., 2021;Pascoal et al., 2018;Wong et al., 2018). Here, we use^18^F-PI-2620, one of thenewer tau-PET tracers. Although, like all tau tracers,^18^F-PI-2620 does show evidenceof off-target binding, most notably in the anterior cerebellum and in the meninges, it benefitsfrom lower off-target binding in the choroid plexus than the widely-used tracer,^18^F-flortaucipir (AV-1451, T807, Tauvid) (Kroth et al., 2019;Mormino et al.,2021), and improved early-stage detection of pathological tau (Aliaga et al., 2024;Bullich etal., 2022;Kroth et al., 2019;Mormino et al., 2021;Mueller et al., 2020).

Previous studies have quantified abnormal tau by calculating mean tracer uptake in a“meta region of interest (ROI),” capturing a broad range of pathological change(Jack et al., 2017;Maass et al., 2017;Wang etal., 2016), and a multi-stage approach quantifying tau in specific spatiotemporalpatterns based on Braak and Braak neuropathological findings (Braak & Braak, 1991). Several studies demonstrate that temporaland temporoparietal ROIs outperform neuropathological staging approaches and are reliable acrosstau tracers (Jack, Wiste, et al., 2018;Leuzy et al., 2020;Villemagne et al., 2023). In key regions previously evaluated inflortaucipir PET studies (Jack, Wiste, et al.,2018;Leuzy et al., 2021), we use areaunder the receiver operating characteristic curve (AUROC), Gaussian mixture modeling (GMM), andtwo standard deviations above the mean signal in cognitively-unimpaired participants, toidentify cut-points that best classify participants as cognitively-impaired using^18^F-PI-2620 standardized uptake value ratios (SUVRs). We identified cut-points andevaluated their performance in a subgroup of Aβ-positive cognitively-impaired (CI) andAβ-negative cognitively-unimpaired (CU) participants (n = 675). Because ofethnoracial differences in Aβ accumulation (Garrett et al., 2019;O’Bryant et al.,2021;Wilkins et al., 2022), we alsoevaluated cut-point performance across the full spectrum of participants (CI and CU), regardlessof Aβ status (n = 879). We use a multi-ethnoracial cohort spanning a range ofsocioeconomic and comorbidity profiles that are more representative of the older population thanmany other cohorts (Lennon et al., 2022;Raman et al., 2022).

## 2 Materials and Methods

### 2.1 Participants

All participants in the Health and Aging Brain Study - Health Disparities (HABS-HD) providedinformed consent in accordance with the Institutional Review Boards for human research (O’Bryant et al., 2021). Participants fromData Drop 6 of HABS-HD who completed a clinical interview, had useable^18^F-florbetaben and^18^F-PI-2620 PET scans within 1.5 years of theircognitive assessment, passed MRI and PET quality control, had available demographicinformation, and had all regions within the temporal composite ROI pass quality control wereincluded in this study (eFig. 1): 879 participants; 729 CU, 120 with mild cognitive impairment(MCI), and 30 with dementia. Most analyses presented in the main text use a subset of theseparticipants categorized by their Aβ PET status: 675 CU Aβ-negative and 76 CIAβ-positive participants (48 with MCI and 28 with dementia, henceforthAlzheimer’s disease; AD). HABS-HD is a collaboration among 18 universities across theUnited States, led by Dr. Sid O’Bryant. All imaging is performed at the University ofNorth Texas Health Science Center, Fort Worth, Texas. Experimental methods, enrollment,inclusion, and exclusion criteria for HABS-HD have been described previously (O’Bryant et al., 2021). Race and ethnicitywere self-identified: 186 were Hispanic/Latino(a) (henceforth Hispanic), 209 were non-HispanicAfrican American/Black (henceforth NHB), and 280 were non-Hispanic White (henceforth NHW)(Table 1).

**Table 1.** Participant demographics by cognitive diagnostic status.

	Aβ- CU (N = 599)	Aβ+ MCI (N = 48)	Aβ+ AD (N = 28)	Overall (N = 675)	p-value
Age	64.62 ± 8.12	70.73 ± 9.33	72.25 ± 9.81	65.37 ± 8.54	<0.001
Sex (%F)	391 (65%)	25 (52%)	13 (46%)	429 (64%)	0.03
Ethnoracial group					0.96
Hispanic	167 (28%)	12 (25%)	7 (25%)	186 (28%)	
Non-Hispanic Black	183 (31%)	17 (35%)	9 (32%)	209 (31%)	
Non-Hispanic White	249 (41%)	19 (40%)	12 (43%)	280 (41%)	
Education (y)	14.41 ± 3.51	13.65 ± 3.68	14.00 ± 4.30	14.34 ± 3.56	0.29
Global Aβ	0.98 ± 0.04	1.34 ± 0.19	1.37 ± 0.22	1.02 ± 0.14	<0.001
Centiloids	4.64 ± 7.04	60.97 ± 30.37	66.70 ± 35.61	11.22 ± 22.40	<0.001
Hippocampal vol	3268.42 ± 368.71	3003.53 ± 369.56	2624.93 ± 652.50	3223.64 ± 408.03	<0.001
*APOE* 4+	132 (22%)	25 (52%)	14 (50%)	171 (25%)	<0.001
Dx hypertension	401 (67%)	33 (69%)	21 (75%)	455 (67%)	0.66
Dx diabetes	154 (26%)	17 (35%)	6 (21%)	177 (26%)	0.28
Dx dyslipidemia	418 (70%)	34 (71%)	20 (71%)	472 (70%)	0.97
BMI	31.38 ± 6.83	31.06 ± 9.40	29.94 ± 7.38	31.30 ± 7.06	0.11
MMSE total	28.39 ± 1.77	26.81 ± 2.65	20.82 ± 4.82	27.96 ± 2.57	<0.001
LM II (A+B total)	25.09 ± 7.19	14.77 ± 8.85	3.71 ± 4.85	23.47 ± 8.73	<0.001
DS total	15.29 ± 3.85	13.33 ± 3.49	10.54 ± 4.10	14.95 ± 3.97	<0.001

Values represent mean (± SD) or percent. Group comparison (differences betweendiagnostic cognitive status) p-values were derived using Kruskal-Wallis tests for continuousvariables and chi-square tests for categorical variables (α = 0.05). Type-IIdiabetes: Participants with a medical history of type-II diabetes or blood hemoglobin AIC≥ 6.5 received a diagnosis of type-II diabetes. Hypertension: Participants with acurrent medical history of hypertension or elevated blood pressure (systolic blood pressure≥ 140 mm Hg or diastolic blood pressure ≥ 90 mmHg) across at least two bloodpressure readings received a diagnosis of hypertension. Ten participants were missing BMIdata, 3 missing MMSE data, 2 missing Digit Span data, and 2 missing hippocampal volume.Centiloids were calculated using this equation: CL = (159.08 ×SUVR_FBB_) − 151.65 based on ADNI3 processing for FBB (Kolibash et al., 2020).

Abbreviations: Dx = diagnosis, Hippocampal Vol = hippocampal volume, BMI= body mass index, MMSE = Mini Mental State Examination, LM-II =Logical Memory-II, DS = Digit Span.

### 2.2 Cognitive diagnosis

Consensus cognitive diagnoses were established by trained clinicians (S1) and as describedpreviously (O’Bryant et al., 2021).

### 2.3 Acquisition of PET and MRI data

T1-weighted whole-brain volumetric spoiled Magnetization-Prepared Rapid Gradient (MPRAGE)scans were acquired (3T Siemens Magnetom Skyra, voxel size 1.1 x 1.1 x 1.2 mm, n = 46;3T Siemens Magnetom Vida system, voxel size was 1 mm x 1 mm x 1, n = 833). MRI imageswere bias corrected using the Advanced Normalization Tools (ANTS) with parameters ofbspline-fitting = [200], shrink factor = 1, and convergence = [50 x 50 x45 x 40, 0]. FreeSurfer version 5.3 was used for cortical parcellation and segmentation (https://surfer.nmr.mgh.harvard.edu/).We evaluated cortical thickness quality-control (QC) outcomes across all ROIs included in thisstudy by assessing the frequency of QC failures within each ethnoracial group (S2). Aβ-and tau-PET scans were acquired on one of two identical Siemens Biograph Vision 450 scanners atthe University of North Texas Health Science Center. Tau-PET imaging (5 mCi +/- 10%^18^F-PI-2620 radiotracer injection; emission scan from 45-75 minutes post-injection;six 5-minute frames) and Aβ-PET imaging (8.1 mCi +/- 10%^18^F-florbetaben (Neuraceq); 20-minute emission scan from 90 minutes post-injection;four 5-minute frames) shared an identical reconstruction protocol (8 iterations/5 subsets withtime of flight on, a 440 x 440 matrix, and an all-pass filter).

### 2.4 Image analysis

T1 MRPAGE MRI,^18^F-PI-2620, and^18^F-florbetaben PET data used for thepreparation of this manuscript are stored, managed, and processed by the University of SouthernCalifornia Laboratory of Neuroimaging. We analyzed the scans using the ADNI3 protocol for^18^F-florbetaben (S. Landau et al.,2011) and an in-house analysis protocol for^18^F-PI-2620 PET. Detailedacquisition and processing methods are outlined in S3.

### 2.5 ^18^ F-florbetaben PET

FreeSurfer-defined regions (frontal, anterior/posterior cingulate, lateral parietal, lateraltemporal cortex; whole cerebellum reference) were used to obtain global Aβ SUVRs, with aglobal SUVR ≥1.08 indicating Aβ positivity (S3).

### 2.6 ^18^ F-PI-2620 PET

Tau-PET summary mean SUVRs were derived from FreeSurfer-defined regions and normalized to thegray matter of the inferior cerebellum (S3, eFig. 2). To check for inflation of SUVRs due tooff-target signal from the meninges and venous sinuses, we also extracted median SUVR measuresfor all ROIs (S4). All median and mean SUVRs were highly correlated (all*r*values ≥0.96), suggesting minimal influence of off-target signal on mean SUVRs. Themaximum time between T1 and^18^F-PI-2620 PET was 17 days.

### 2.7 ^18^ F-PI-2620 image processing for voxelwise analysis

Each participant’s T1-MRI was warped to MNI152 template space using a non-lineartransformation (FSL FNIRT).^18^F-PI-2620 SUVR images were created by applying thesetransformation parameters to the corresponding^18^F-PI-2620 SUVR image in T1 space(S5).

## 3 Statistical Analyses

### 3.1 Cognitive diagnostic group differences

Differences in demographics, comorbid conditions, global Aβ, and cognitivemeasurements across diagnostic groups were assessed using Kruskal-Wallis tests followed byDunn’s post-hoc tests for continuous variables and chi-square tests for categoricalvariables (Table 1).

### 3.2 Region of interest selection

SUVRs in regions known to be affected by AD and frequently-examined in flortaucipir tau-PETstudies were evaluated (Botha et al., 2018;Cho et al., 2018;Dodich et al., 2020;Guo etal., 2020;Jack et al., 2017,^2019^;Leuzy et al., 2023;Lowe et al.,2018,^2018^;Maass et al., 2017;Ossenkoppele et al., 2018;Schöll etal., 2016;Schwarz et al., 2018;Wang et al., 2016;Weigand et al., 2022): entorhinal cortex, fusiform gyrus, inferior andmiddle temporal gyri, parahippocampal cortex, amygdala, and hippocampus. We also evaluated tausignal in the posterior cingulate and lateral parietal regions. Two composite ROIs were createdusing a volume-weighted average of the mean uptake in 1) a temporal composite, which includedall regions listed above except for posterior cingulate and lateral parietal cortex, and 2)regions with the highest positive correlation with diagnosis in this sample: entorhinal cortex,parahippocampal cortex, and amygdala (eFig. 3; henceforth the “medial temporal”ROI).

### 3.3 Voxelwise associations between diagnostic groups

**Fig. 1.**
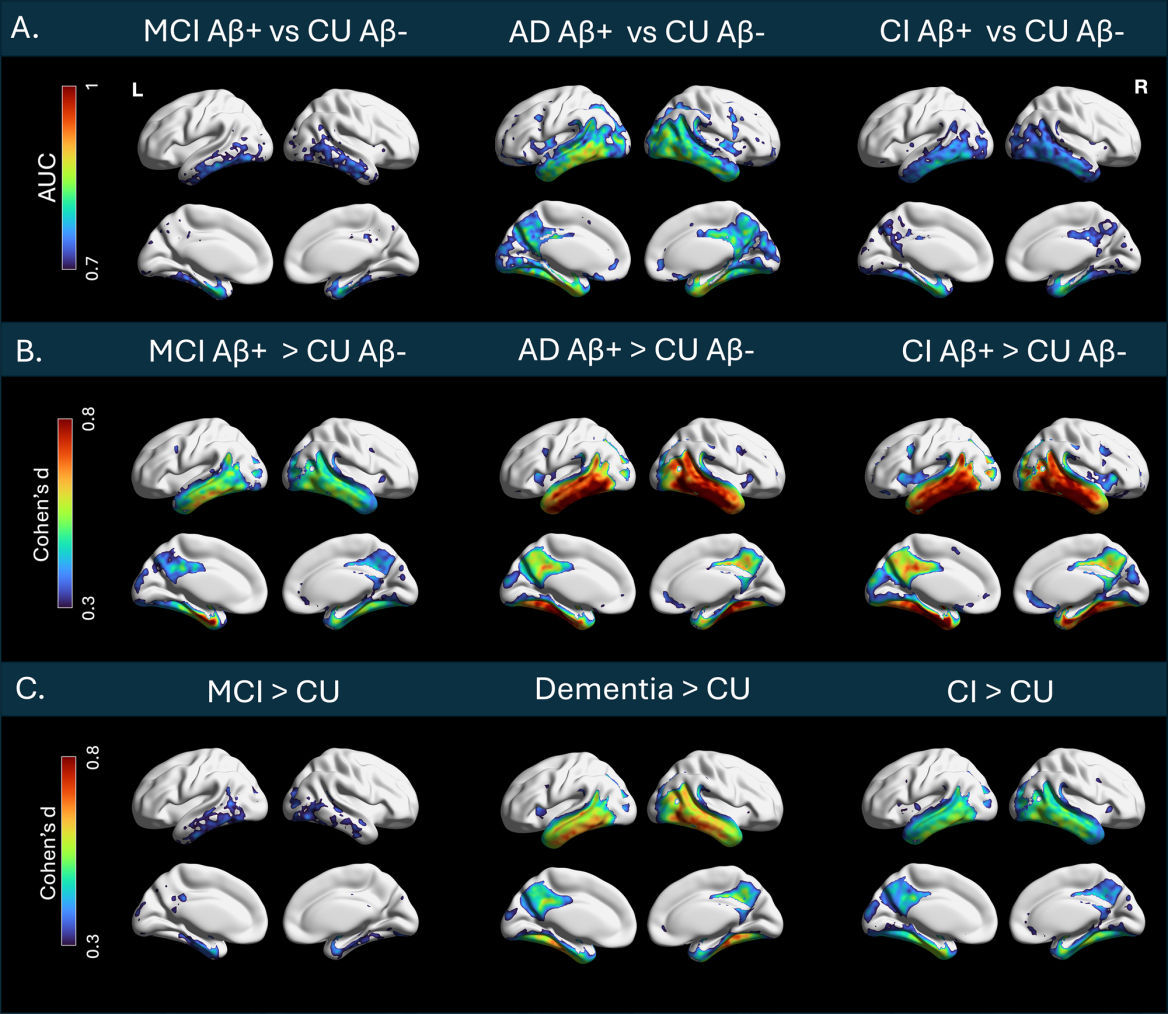
Voxelwise comparisons between diagnostic cognitive status. All results were thresholded atpFWE<0.05 at the cluster level and p < 0.001 at the voxel level before beingrendered on the surface brain. T-maps were transformed to effect-size maps usingCohen’s d. (A) Voxelwise AUC values across diagnostic groups. Regions with the highestAUC when distinguishing CU from MCI were the parahippocampal cortex, entorhinal cortex,amygdala, and fusiform gyrus. These regions also had the highest AUC when distinguishing CUfrom AD, in addition to the inferior and middle temporal gyri, cingulate, and precuneus.These regions remained consistent but with lower AUC when distinguishing between CU and CIparticipants. MCI Aβ+ (n = 48), AD Aβ+ (n = 28), CIAβ+ (n = 76), CU Aβ- (n = 599). (B) Voxelwise t-testscomparing tau in CU Aβ- and CI Aβ+. Both MCI and AD participants showedmore uptake in the parahippocampal cortex, entorhinal cortex, and fusiform gyrus compared toCU participants. AD participants showed more binding in the middle temporal gyri, cingulate,and precuneus compared to CU and MCI groups. (C) Voxelwise t-tests comparing tau in the fullspectrum of participants irrespective of Aβ status. Patterns reveal similar, albeitsmaller differences in tau deposition. MCI (n = 120), dementia (n = 30), CI (n= 150), and CU (n = 729).

To assess whole-brain^18^F-PI-2620 patterns between diagnostic groups, voxelwiseanalyses were conducted using SPM-12 independent sample t-tests. Resulting T-maps weretransformed to effect-size maps (Cohen’s d) for ease of interpretation (Fig. 1).

### 3.4 Voxelwise ROC analysis

Voxelwise ROC analyses were performed by diagnostic group using the VoxelStats toolbox (Mathotaarachchi et al., 2016) to derive voxelwiseAUC values (Fig. 1).

### 3.5 Best-performing ROI and cut-point calculations

Tau cut-points were assessed using two classification methods: (A) AUROC analyses and (B)calculation of two standard deviations above the mean (2SD+) SUVR value in CUAβ-negative participants for the best-performing ROI identified in method A. Weperformed AUROC analyses in two ways: 1) using the mean SUVR in each ROI and 2) using theprobabilities derived by applying a two-component (bimodal) Gaussian mixture model (GMM) toeach ROI to estimate the probability of each SUVR belonging to the second Gaussian(‘abnormal’ or tau-positive) distribution. We identified the best-performing ROIand cut-point as that having the highest Youden index (sensitivity + specificity-1). AYouden index of at least 0.6 with a sensitivity or specificity of at least 0.5 was consideredacceptable (Chen et al., 2015). We ran 1000stratified bootstrap replicates to obtain a 95% confidence interval on the cut-points.Accuracy, sensitivity, specificity, Youden Index, and cut-points were calculated for eachROI.

### 3.6 Defining cut-points across the Aß spectrum

The same AUROC analyses were performed in the full spectrum of participants regardless oftheir Aβ status. Continuous global Aβ was included in these AUROC analyses(eTable 2).

### 3.7 Evaluating the performance of the cut-point across ethnoracial groups

Participants within each ethnoracial group were classified as tau positive or negative iftheir SUVR value in the best-performing ROI was above or below the identified cut-point forthat region. We used the cut-point with the highest Youden index to classify participants astau positive or negative. True positives were classified as any participant with a diagnosis ofMCI or AD whose SUVR value in the best-performing ROI was greater than the identifiedcut-point. Additionally, AUROC analyses were conducted in each ethnoracial group separately(eTable 6).

## 4 Results

### 4.1 Cognitive diagnostic group differences

Group differences are shown inTable 1.

### 4.2 Voxelwise differences between diagnostic groups

Both MCI and AD participants showed more tau uptake in the entorhinal cortex, fusiform gyrus,and parahippocampal cortex compared to CU participants. AD participants additionally showedmore binding in the inferior and middle temporal gyri, cingulate, and precuneus compared to CUand MCI groups (Fig. 1). Comparisons betweendiagnostic groups irrespective of Aβ status reveal similar, albeit weaker taudifferences. Mean voxelwise^18^F-PI-2620 signal by diagnostic group is shown ineFigure 4.

### 4.3 Voxelwise SUVR AUC

The maximum voxelwise AUC across group comparisons was 0.88. Regions with an AUC > 0.7for all diagnostic comparisons included the fusiform gyrus, parahippocampal cortex, amygdala,and middle and inferior temporal gyri. For CU versus AD, the cingulate, precuneus, frontal, andoccipital regions also exceeded an AUC of 0.7 (Fig.1).

### 4.4 Best-performing ROI and cut-point for classifying cognitive diagnostic status

AUROC results showed that the medial temporal ROI had the highest Youden index for alldiagnostic comparisons, and was acceptable for differentiating CU from MCI, AD, and CI. Whenseparating CU from both AD and CI, the cut-point for this ROI was 1.26 and when separating CUfrom MCI it was 1.28 (Table 2). SUVRs in allregions successfully distinguished CU from AD. See eTable 1 for comprehensive results.

**Table 2.** Results of classification methods.

Best-performing ROI: MTL composite	SUVR AUROC			AUROC (predicted probabilities)			2 SD+		
	CU vs. MCI	CU vs. AD	CU vs. CI	CU vs. MCI	CU vs. AD	CU vs. CI	CU vs. MCI	CU vs. AD	CU vs. CI
Optimal cut-point	1.28	1.26	1.26	—	—	—	1.32	1.32	1.32
Youden	0.62	0.68	0.63	0.61	0.66	0.62	0.52	0.62	0.55
Sensitivity	0.67	0.75	0.71	0.71	0.68	0.72	0.54	0.64	0.58
Specificity	0.95	0.93	0.92	0.90	0.98	0.90	0.97	0.98	0.98
Accuracy	0.93	0.92	0.90	0.89	0.97	0.88	0.94	0.96	0.93
AUC	0.81	0.84	0.82	0.87	0.85	0.85	-	-	-

Performance metrics for the MTL composite (volume weighted average of the entorhinal,parahippocampus, and amygdala SUVRs), the best-performing ROI in the AUROC when using meanSUVRs and predicted probabilities from the GMMs. All CU participants were Aβ- and allCI participants were Aβ+. Results for the AUROC in the full spectrum ofparticipants, regardless of Aβ status, is in eTable 4.

### 4.5 AUROC using predicted probabilities from GMM

AUROC results using predicted probabilities (eTable 2) also showed that the medial temporalROI had the highest Youden index across diagnostic classifications. AUROC using predictedprobabilities in all regions successfully distinguished CU from AD.

### 4.6 2SD+

Using the 2SD+ method, a cut-point of 1.32 was determined for the medial temporal ROI(Table 2), with the only acceptable Youdenindex distinguishing CU versus AD (>0.60).

### 4.7 Tau cut-points without regard to Aß positivity

Of the 879 participants, 204 did not meet the Aβ-stratification criteria, including130 Aβ-positive CU participants (14% Hispanic, 14% NHB, and 23% NHW), 72Aβ-negative MCI participants, (73% Hispanic, 53% NHB, and 51% NHW), and 2Aβ-negative participants with dementia (1 Hispanic; 1 NHW). Aβ status bydiagnostic and ethnoracial group is shown in eTable 3.

In the full spectrum (eTable 4), AUROC analyses showed that continuous global Aβ bestdifferentiated CU from dementia, with the highest Youden index (0.76). Discrimination for allmeasures was weaker for CU versus MCI and CU versus CI, with Youden indexes below 0.35,suggesting that tau cut-points are effective in explaining cognitive status predominantly inAβ-positive individuals.

### 4.8 Differences in cut-point performance across ethnoracial groups

Demographics by ethnoracial group are shown inTable 3. Mean SUVR by ethnoracial group can be found in eTable 5. The 1.26 cut-pointperformed acceptably in Hispanic and NHW participants, but not in NHB participants (Table 4). When AUROC were fit in each ethnoracialgroup separately, the medial temporal composite consistently showed the highest Youden index(eTable 6).

**Table 3.** Participant demographics by ethnoracial group.

	Hispanic (N = 186)	Non-Hispanic Black (N = 209)	Non-Hispanic White (N = 280)	Overall (N = 675)	p-value
Aβ- CU	167 (89%)	183 (88%)	249 (89%)	599 (89%)	-
Aβ+ MCI	12 (7%)	17 (8%)	19 (7%)	48 (7%)	-
Aβ+ AD	7 (4%)	9 (4%)	12 (4%)	28 (4%)	-
Age	64.14 ± 8.49	61.85 ± 7.46	68.81 ± 8.05	65.37 ± 8.54	<0.001
Sex (%F)	128 (69%)	141 (68%)	160 (57%)	429 (64%)	0.03
Education (y)	11.32 ± 4.04	14.98 ± 2.71	15.87 ± 2.36	14.34 ± 3.56	<0.001
Global Aβ	1.03 ± 0.15	1.02 ± 0.11	1.03 ± 0.16	1.02 ± 0.14	0.72
Centiloids	11.55 ± 23.30	9.84 ± 17.53	12.02 ± 24.92	11.22 ± 22.40	0.72
Hippocampal vol	3234.42 ± 426.61	3170.94 ± 388.93	3255.86 ± 406.81	3223.64 ± 408.03	0.10
*APOE* 4+	32 (17%)	67 (32%)	72 (26%)	171 (25%)	0.001
Dx hypertension	119 (64%)	161 (77%)	175 (63%)	455 (67%)	0.001
Dx type II diabetes	68 (37%)	59 (28%)	50 (18%)	177 (26%)	0.28
Dx dyslipidemia	147 (79%)	123 (59%)	202 (72%)	472 (70%)	<0.001
BMI	30.93 ± 5.82	33.52 ± 8.38	29.87 ± 6.29	31.30 ± 7.06	<0.001
MMSE total	27.02 ± 2.91	27.74 ± 2.66	28.75 ± 1.94	27.96 ± 2.57	<0.01
LM-II A+B total	21.83 ± 8.20	21.20 ± 8.19	26.24 ± 8.72	23.47 ± 8.73	<0.001
DS total	12.46 ± 3.39	14.94 ± 3.55	16.61 ± 3.76	14.95 ± 3.97	<0.001

Group comparison (differences between ethnoracial groups) p-values were derived usingKruskal-Wallis tests for continuous variables and chi-square tests for categorical variables(α = 0.05). Continuous variables are listed as mean (±standarddeviation). Categorical variables are listed as number (percent). Ten participants weremissing BMI data, 3 missing MMSE data, 2 missing Digit Span data, and 2 missing hippocampalvolume. Centiloids were calculated using this equation: CL = (159.08 ×SUVR_FBB_) − 151.65 based on ADNI3 processing for FBB (Kolibash et al., 2020).

Abbreviations: Dx = diagnosis, Hippocampal Vol = hippocampal volume, BMI= body mass index, MMSE = Mini Mental State Examination, LM-II =Logical Memory-II, DS = Digit Span.

**Table 4.** Cut-point performance across ethnoracial groups.

	AUROC: 1.26			2 SD+: 1.32		
Method & cut-point	Hispanic	NHW	NHB	Hispanic	NHW	NHB
Youden	0.78	0.66	0.45	0.71	0.55	0.45
Sensitivity	0.84	0.74	0.54	0.74	0.58	0.46
Specificity	0.94	0.92	0.91	0.97	0.97	0.98
Accuracy	0.93	0.90	0.86	0.95	0.93	0.92

In CU Aβ- and CI Aβ+ participants, we tested the tau cut-point of1.26 to classify participants as cognitively impaired. The 2SD+ cut-point was 1.32(2SD+ above the mean SUVR in the medial temporal ROI of cognitively-unimpairedparticipants).

## 5 Discussion

Using^18^F-PI-2620 we have identified an ROI and corresponding cut-point thatdifferentiates cognitive status in a multi-ethnic/racial cohort. The medial temporal compositehad the highest Youden index across all diagnostic classifications, indicating that thiscomposite was sensitive to the earliest clinically-relevant tau accumulations in this sample.The high specificity of tau in this region suggests that here: 1) the presence of tau is notoverestimated and 2) the presence of tau is a good indicator of cognitive impairment. However,studies have shown that tau accumulation in the entorhinal cortex slows as disease progresses(Jack, Wiste, et al., 2018;Villemagne et al., 2021), necessitating futureresearch to evaluate the effectiveness of this ROI in predicting longitudinal changes incognition and tau accumulation. The sensitivity observed across all ROIs was notably low,particularly in NHB participants. This may indicate the influence of other factors thatcontribute more to cognitive impairment in NHB participants than tau alone (Harrison et al., 2025). When all participants wereincluded (irrespective of Aβ status) Aβ was more effective than tau atdistinguishing cognitive impairment. This suggests that 1) in our multi-ethnic cohort,consistent with prior work (Robinson et al.,2024), tau in the absence of Aβ positivity does not meaningfully explaincognitive impairment and 2) tau positivity using these cut-offs should only be used to identifyclinically-meaningful tau levels in Aβ-positive individuals.

In past work in NHW participants, 54% of MCI patients and 81.4% of dementia patients wereAβ positive, similar to the 49% and 92% Aβ-positive NHW MCI and dementia patientswe found here, respectively. However, also as reflected in our work, Aβ-positivity ratesmay vary by ethnoracial group (Jang et al.,2024). In cognitively-impaired Aβ-positive individuals, the presence of tauwithin the normal range represents an atypical disease trajectory, with approximately20–40% of Aβ-positive individuals remaining tau-negative depending on cohortcomposition and diagnostic criteria (Josephs et al.,2022;S. M. Landau et al., 2024;Spina et al., 2021) consistent with the 30% taunegativity in Aβ-positive cognitively-impaired participants in our study. For instance,past work using flortaucipir found that tau positivity in the entorhinal cortex was 80.2% inAβ-positive dementia patients, 50.2% in Aβ-positive MCI patients, and 6.3% incognitively-unimpaired people (Ossenkoppele et al.,2021). Our tau-positivity rate in Aβ-positive dementia patients was similar at75%. However, tau positivity was higher in our sample for Aβ-positive MCI patients (67%)and CU participants without respect to Aβ status (12%). The relatively high number ofAβ-negative MCI cases among Hispanic and NHB participants further supports thepossibility that non-amyloid, non-tau mechanisms may play a role in disease progression amongthese groups, warranting further investigation. Future work should also evaluate the extent towhich the region of interest used, the ethnoracial composition of the sample, or the tracer usedaffects tau-positivity status.

Several studies have found that NHB individuals who achieve low scores on neuropsychologicalexams have lower Aβ-positivity rates than NHW participants (Carrillo & Mahinrad, 2024;Morris et al., 2019;Raman etal., 2021), suggesting that cognitive impairment or clinical symptoms may be morestrongly influenced by non-AD pathways, including cerebrovascular injury—particularlygiven the higher prevalence of cardiovascular disease in NHB populations (Hines et al., 2023). However, lowerneuropsychological test scores may reflect socioeconomic and educational disparities rather thanAD pathology, potentially resulting in overdiagnosis of NHB individuals with MCI when a singletime point is considered (Raman et al., 2021).To address this, we compared the cut-point performance between participants with highereducation and those with a high-school degree and observed no significant difference in AUROCbetween the groups (eTable 7). NHB participants in the current study are also, on average,younger than the Hispanic and NHW participants, which may increase the likelihood that cognitiveimpairment is attributable to pathologies other than Aβ and tau. When available, futureresearch in diverse populations should incorporate additional information about comorbiditiesand other neuropathologies to determine whether these factors influence cut-point values ortheir utility.

Although use of the 2SD+ method resulted in a more conservative cut-point and similaraccuracy to ROC, it resulted in a Youden index that was lower for all diagnostic groupcomparisons. The AUROC method incorporates clinical relevance (through sensitivity andspecificity) to determine the optimal cut-points. While the 2SD+ method provides astricter threshold, AUROC may be preferable for identifying clinically-meaningful distinctionsbetween groups. We recognize that using the Youden index prioritizes overall diagnostic accuracyby maximizing the sum of sensitivity and specificity but may overlook context-specificpriorities where the costs of false positives and false negatives differ.

## 6 Strengths and Limitations

The strengths of this study include the multi-ethnoracial composition of the sample and thenovelty of^18^F-PI-2620. Although the heterogeneity of the sample affectedsensitivity, the results point to a gap in knowledge as to what other factors influence theclinical phenotype in more diverse samples. We used the cut-point obtained from the full modeland applied it to each ethnoracial group, acknowledging limitations due to key differencesbetween the three groups. However, we believe this approach demonstrates the challenges ofdefining pathological protein levels within heterogeneous samples. Additionally, similar resultswere observed when cut-points were calculated independently for each group, though the smallersample sizes in some groups limit the robustness and generalizability of these findings. Ofnote, the prevalence of cognitively-impaired Hispanic participants in this sub-sample was lowerthan in the larger HABS-HD sample. In the full spectrum (regardless of Aβ status), weincluded 72 new Aβ-negative participants with MCI across ethnoracial groups but onlyadded two Aβ-negative participants with dementia. As a result, nearly all participantswith dementia were Aβ positive, which likely explains why Aβ status was a strongerpredictor of dementia than tau in our full sample, despite previous research suggesting that taucorrelates more closely with cognitive status than Aβ. Finally, the cross-sectionalnature of our study should be considered when interpreting the results.

## 7 Conclusions

We have established tau cut-points for cognitive diagnosis using^18^F-PI-2620. Ourdata demonstrate the utility of the medial temporal ROI to identify clinically relevant taulevels in Hispanic and NHW individuals.

## Supplementary Material

Supplementary Material

## Data Availability

Data used in preparation of this manuscript preparation are stored, managed, and processed bythe University of Southern California (USC) Laboratory of Neuroimaging (https://ida.loni.usc.edu/). All data are availableupon request.
