# Supplementary material for: Establishing tau-PET cut-points for cognitive diagnosis with 18 F-PI-2620 in a multi-ethnoracial cohort
Source: Imaging Neurosci (Camb). 2025 Jun 16;3:IMAG.a.41. doi: 10.1162/IMAG.a.41 (PMC12319742; doi:10.1162/IMAG.a.41)
Supplement: Supplementary Material [file imag.a.41_supp.pdf]

Table of Contents

S1. Cognitive Diagnosis 1

eFigure 1. Participant inclusion criteria. 2

S2. Evaluation of region of interest segmentations by ethnoracial group. 2

S3. PET Acquisition and processing 2

eFigure 2. Gray matter of the inferior cerebellum reference region 4

S4. ROI contamination testing 4

S5. 18F-PI-2620 image processing for voxelwise analysis & contamination testing 4

eFigure 3. Composite ROIs used in cut point analysis. 5

eFigure 4. Mean 18F-PI-2620 signal across diagnostic groups 5

eTable 1. AUROC results using regional and composite SUVRs 6

eTable 2. AUROC results using predicted probabilities derived from Gaussian mixture models (GMM). 7

eTable 3. Amyloid & tau status by cognitive and ethnoracial group 9

eTable 4. AUROC results using SUVRs in the full spectrum of participants, irrespective of amyloid status 10

eTable 5. Mean ± SD of SUVRs across ethnoracial and cognitive groups in 675 participants 11

eFigure 5. SUVR Distributions by Diagnosis and Ethnoracial group 12

eTable 6. AUROC results fit in each ethnoracial group separately 13

eTable 7. Performance of the 1.26 cut-point in participants with high and low education 14

S1. Cognitive Diagnosis

Cognitive diagnosis was established by a consensus among trained clinicians[1]. **Normal cognition:** clinical dementia rating scale sum of boxes score = 0, and cognitive test scores considered broadly within normal limits. Of note, if a participant had an isolated poor neuropsychological test performance, in the absence of any cognitive complaints and functional decline, they were considered to have normal cognition. **MCI:** complaint of cognitive change (self or other), CDR sum of boxes score of 0.5-2.0, and performance at or below 1.5 standard deviations below Z-score adjusted norms on at least one cognitive test. **Dementia:** complaint of cognitive change (self or other), CDR sum of boxes score ≥2.5, and a cognitive test score at or below two standard deviations below the mean on two or more cognitive domains.

eFigure 1. Participant inclusion criteria.

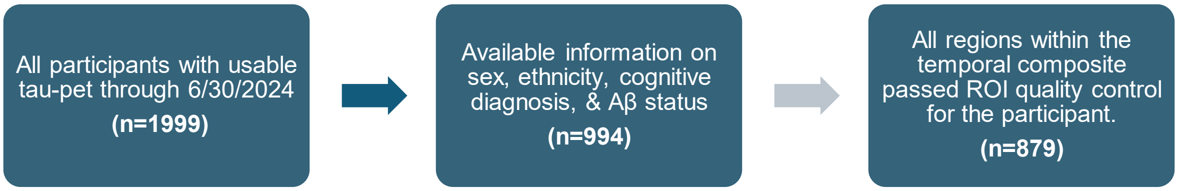

We only included participants who passed visual quality control (QC) of the FreeSurfer segmentations for all the regions of interest (ROIs) included within the temporal composite. Before lab members are approved to quality check PI-2620 tau scans in the HABS-HD cohort, they must complete a training set and take a reliability test (Cohen's kappa ≥ 0.60) using a set of scans that have already been quality checked and normed by a team of experienced raters. We also evaluated the QC outcomes for all ROIs included in the temporal composite (entorhinal, fusiform, inferior temporal, parahippocampal, amygdala, middle temporal, hippocampus). Specifically, we assessed the frequency of QC failures recorded for each ROI within different ethnoracial groups. We conducted Fisher's exact tests for each ROI. These tests evaluated whether the frequency of QC failures differed significantly between ethnoracial groups. We found no statistically significant associations between ROI QC failure rates and ethnoracial group for any of the ROIs in the temporal composite ($p>0.05$).

S2. Evaluation of region of interest segmentations by ethnoracial group.
To ensure that the cut point results across ethnoracial groups we not influenced by differences in segmentation quality for our regions of interest, we again evaluated the QC outcomes for all ROIs included in our analyses (entorhinal, fusiform, inferior temporal, parahippocampal, posterior cingulate, middle temporal, hippocampus, and lateral parietal). Specifically, we assessed the frequency of QC failures recorded for each ROI within different ethnoracial groups. We conducted Fisher's exact tests for each ROI. These tests evaluated whether the frequency of QC failures differed significantly between ethnoracial groups. We found no statistically significant associations between QC failure rates and ethnoracial group for any of the ROIs used ($p>0.05$). These findings suggest that methodological issues related to cortical thickness QC are unlikely to differentially influence the calculation of SUVR values in this sample of HABS-HD participants.

S3. PET Acquisition and processing
PI2620 PET: Participants received an intravenous bolus injection of 5 mCi bolus ± 10% of PI-2620. A CT scan was acquired for attenuation correction. Between 45 and 75 minutes post-injection, a dynamic PET scan was acquired, consisting of six 5-minute frames (totaling 30 minutes). PET images were reconstructed after acquisition using an iterative algorithm (8 iterations, 5 subsets), with time-of-flight on, a matrix of 440 × 440 × 119, zoom of 2, and an all-pass filter on. Unprocessed, reconstructed tau PET scans were obtained from the Laboratory of Neuro Imaging (LONI) Image & Data Archive (IDA). To correct for participant motion, all frames were co-registered to the first frame using SPM12's reslice and realign tool and motion-corrected frames were then averaged to generate a single static 3D PET image. Because prior studies show 18F-PI-2620 off-target binding in the vermis and anterior lobe of the cerebellum[2,3], we created a more restricted reference region by excluding more of the superior portion of the cerebellum gray matter[4]. To create this reference region, we used the SUIT cerebellar atlas[5,6] in Montreal Neurological Institute space and thresholded the standardized intensity values of the atlas mask at 8, resulting in a mask that delineated the inferior cerebellum while excluding areas above the bilateral Crus I lobule. Using FSL FNIRT, we non-linearly warped this mask to each participant's bias-corrected, skull-stripped T1-weighted MRI scan and multiplied the SUIT mask

by the individual participants' FreeSurfer-derived (version 5.3) cerebellar gray matter masks in T1 native space to derive each individual's native MR space reference region.

For ROI PET analyses, we then used FSL FLIRT to linearly align each participant's PET scan to the T1-weighted MR images (6 degrees of freedom) using a mutual information cost function. The inverse coregistration matrices were applied to the ROIs and reference regions to bring them into native PET image space, where our region of interest analyses were conducted. We calculated SUVRs for each region by dividing mean signal in the ROI by mean signal in the reference region.

Please see section **S5** below for information on 18F-PI-2620 image processing for voxelwise analysis.

Florbetaben PET: Participants received an intravenous bolus injection of 8.1 mCi (±10%) of [^{18}F]florbetaben. A low-dose CT scan was acquired for attenuation correction. At 90 minutes post-injection, a dynamic PET acquisition was performed consisting of four 5-minute frames (totaling 20 minutes). Image reconstruction was performed using the same parameters described for the Siemens Biograph Vision 450 scanner in the PI-2620 protocol. Unprocessed, reconstructed amyloid PET scans were obtained from the LONI IDA. Scans were first motion corrected by co-registering each frame to the individual's first frame using SPM12's re-slice and realign tools. Motion-corrected frames were then averaged to generate a static PET image. Consistent with the ADNI3 florbetaben processing protocol, averaged images were resampled to 1.5 mm isotropic voxels and smoothed with a 6.5 mm full-width-at-half-maximum (FWHM) in-plane Gaussian kernel and 5.5 mm FWHM through-plane, resulting in an approximate resolution of ~8 mm. FreeSurfer version 5.3.0 was used to segment each participant's MRI T1-weighted scan into frontal, lateral parietal, anterior/posterior cingulate, and lateral temporal ROIs[7].These ROIs were then applied to the florbetaben PET scan. We also used FreeSurfer to delineate the whole cerebellum reference region for florbetaben. Each participant's PET scan was linearly co-registered to the T1-weighted MR images (6 degrees of freedom) FSL FLIRT with a mutual information cost function. The inverse coregistration matrices were applied to the ROIs and reference regions to bring them into native PET image space, where our analyses were conducted. We calculated SUVRs for each region by dividing mean signal in the ROI by mean signal in the reference region.

eFigure 2. Gray matter of the inferior cerebellum reference region

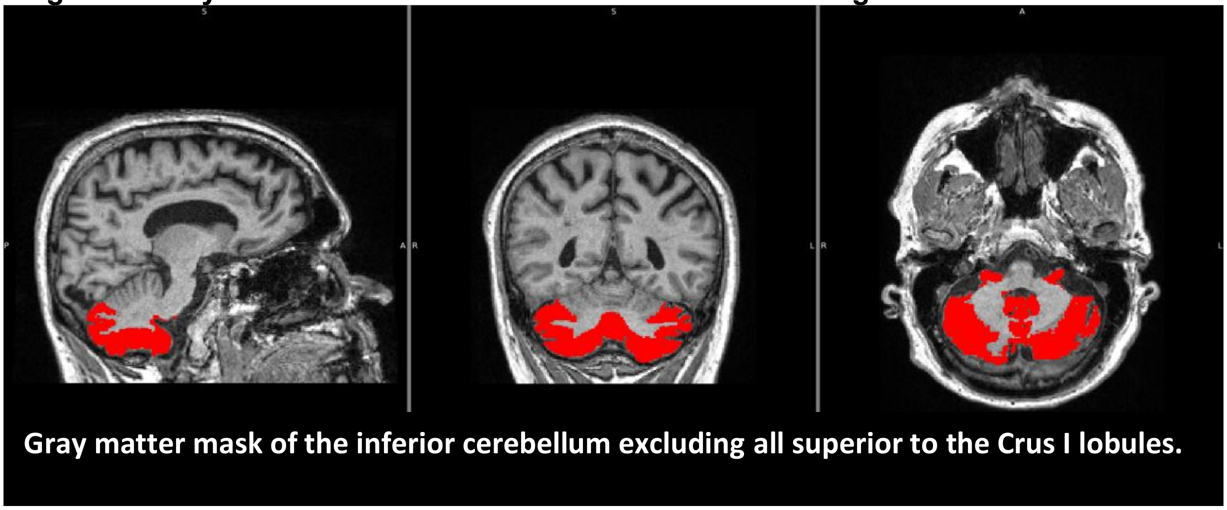

Gray matter mask of the inferior cerebellum excluding all superior to the Crus I lobules.

S4. ROI contamination testing

Median SUVR values are less influenced by outlying data points, such as signal from small amounts of off-target binding, than mean values are. To evaluate whether our results were being influenced by off-target binding, we used FSL to extract median signal in all ROIs and divided those median ROI values by the mean signal in the inferior cerebellum reference region. The reference region masks were separately evaluated for off-target binding and failed if such binding was present. We created the median composite ROIs using a volume-weighted average of the median SUVRs for each region in the composite. All median and mean SUVR values for each ROI were highly correlated, with Pearson correlation coefficients exceeding ≥ 0.96. When predicting cognitive status (CU vs MCI, CU vs AD and CU vs CI), the Youden index and area under the curve (AUC) were higher for the entorhinal, parahippocampal, and amygdala composite when using mean values compared to median values, another indication that the mean SUVRs were measuring clinically important signal. See eTable 1 below for the Youden index and AUC using mean and median SUVRs for the entorhinal, parahippocampal, and amygdala composite ROI.

S5. 18F-PI-2620 image processing for voxelwise analysis & contamination testing

The registration, segmentation, and signal contamination of all ROIs underwent visual quality checks by imaging analysts who had completed amyloid and tau PET quality control training and had demonstrated high inter-rater reliability for passing or failing various analysis steps (Cohen’s kappa ≥ 0.6) when compared with known answers from a training set created by expert raters.

During the ^{18}F-PI-2620 voxelwise PET processing pipeline, each participant’s bias-corrected (using ANTS N4 bias correction) T1 MPRAGE image had non-brain matter removed using FreeSurfer output, and the resulting skull-stripped T1 image was warped to MNI template space using a non-linear transformation (FSL FNIRT). The ^{18}F-PI-2620 SUVR images also were normalized to MNI template space by first linearly coregistering them to the MRI in native space (FSL FLIRT), and then non-linearly transforming them to MNI template space (FSL FNIRT) using the non-linear MPRAGE-to-MNI transformation generated above. Each participant’s binarized, skull-stripped T1-in-MNI space was applied to their corresponding ^{18}F-PI-2620 SUVR image in MNI space to create a skull-stripped SUVR image. For the voxelwise analyses only, each skull-stripped ^{18}F-PI-2620 SUVR image in MNI space was smoothed with a 4 mm isotropic

Gaussian Kernel (SPM12). Visual quality control was conducted on all warped, masked, and smoothed SUVR images before analysis was performed in MNI space.

Explicit mask used in SPM12 voxelwise analyses: To eliminate possible signal from off-target binding from the meninges in the voxelwise analysis, we used *fslmaths* to erode a T1 MNI template brain using a kernel sphere of 6 and the -ero flag. We then multiplied the eroded template brain by a template space gray matter mask so that only voxels not contiguous with the external surface of the cortex would be considered in the voxelwise analyses.

eFigure 3. Composite ROIs used in cut point analysis.

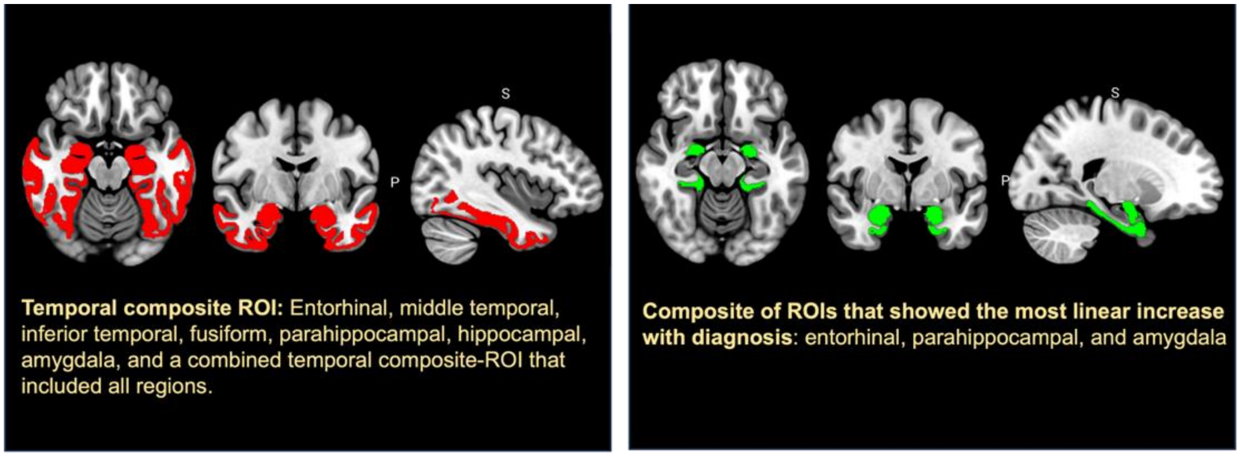

eFigure 4. Mean 18F-PI-2620 signal across diagnostic groups

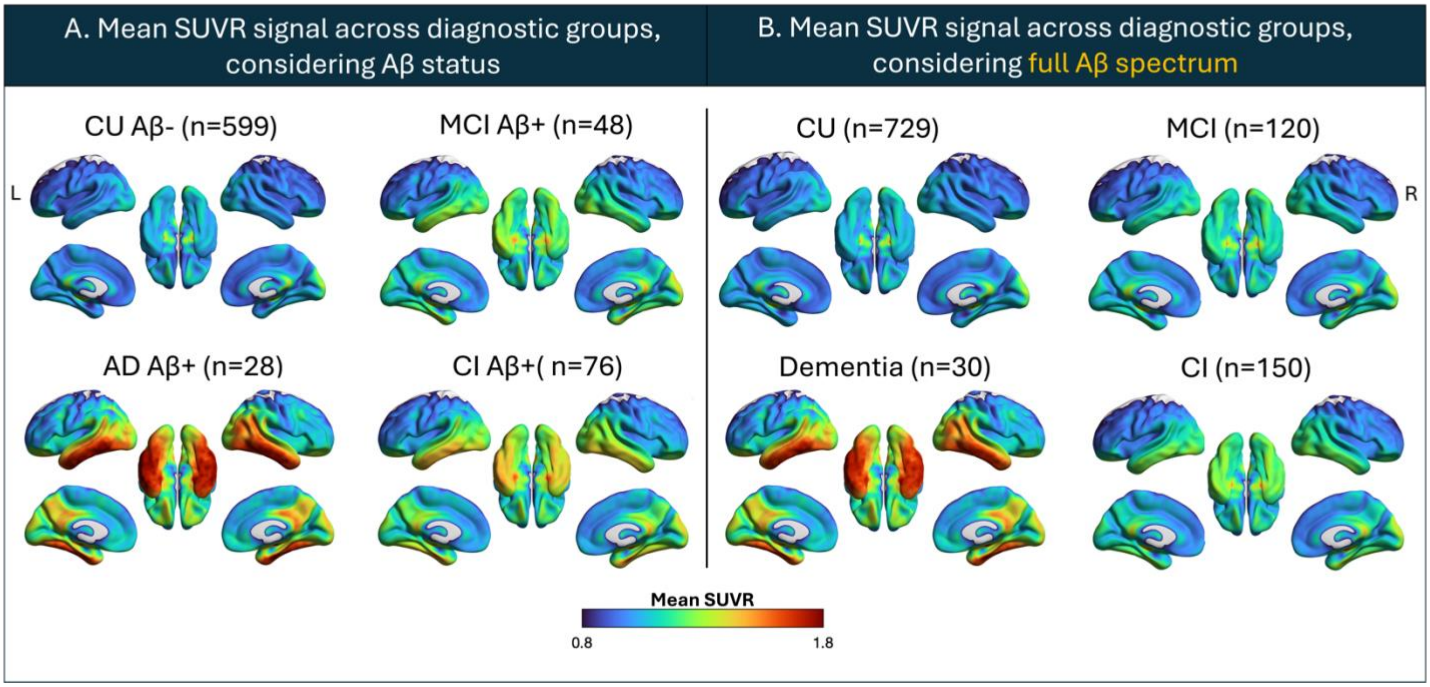

A. Mean SUVR considering participant Aβ status, with groups stratified into CU Aβ -, MCI Aβ +, AD Aβ +, and CI Aβ + individuals. **B.** Mean SUVR across the full diagnostic spectrum without stratification by Aβ status. The color bar represents the 5th (0.8) and 95th (1.8) percentiles for mean SUVRs.

eTable 1. AUROC results using regional and composite SUVRs
Area under the receiver operating characteristic curve (AUROC) analyses were conducted using the "cutpointr" package (R version 4.3.1) with the method function *"maximize_metric"* and the metric function "*Youden*". The medial temporal and temporal composite regions are depicted in eFigure 3.

CU Aβ- (n=599) vs MCI Aβ+ (n=48)

ROI SUVR	Youden	Accuracy	AUC	Cut point	Sensitivity	Specificity
Medial temporal composite (ERC+PHC+AMYG)	0.62	0.93	0.81	1.28	0.67	0.95
Entorhinal	0.56	0.77	0.81	1.39	0.79	0.76
Parahippocampus	0.55	0.89	0.78	1.27	0.65	0.90
Temporal composite	0.54	0.91	0.78	1.20	0.60	0.93
Inferior temporal	0.52	0.88	0.78	1.17	0.62	0.90
Amygdala	0.50	0.84	0.79	1.00	0.65	0.86
Fusiform	0.49	0.80	0.76	1.18	0.69	0.80
Middle temporal	0.46	0.86	0.75	1.15	0.58	0.88
Lateral parietal	0.40	0.88	0.71	1.10	0.50	0.90
Posterior cingulate	0.36	0.71	0.72	1.06	0.65	0.71
Hippocampus	0.32	0.74	0.66	1.17	0.56	0.76

CU Aβ- (n=599) vs AD Aβ+ (n=28)

ROI SUVR	Youden	Accuracy	AUC	Cut point	Sensitivity	Specificity
Medial temporal composite (ERC+PHC+AMYG)	0.68	0.92	0.84	1.26	0.75	0.93
Amygdala	0.67	0.91	0.84	1.03	0.75	0.92
Temporal composite	0.67	0.98	0.85	1.27	0.68	0.99
Inferior temporal	0.66	0.96	0.86	1.26	0.68	0.98
Entorhinal	0.63	0.94	0.84	1.55	0.68	0.95
Middle temporal	0.63	0.97	0.81	1.23	0.64	0.99
Parahippocampus	0.63	0.94	0.83	1.30	0.68	0.95
Fusiform	0.62	0.87	0.87	1.19	0.75	0.88
Lateral parietal	0.59	0.90	0.81	1.10	0.68	0.91
Posterior cingulate	0.55	0.90	0.78	1.12	0.64	0.91
Hippocampus	0.53	0.84	0.80	1.22	0.68	0.85

CU Aβ- (n=599) vs CI Aβ+ (n=76)

ROI SUVR	Youden	Accuracy	AUC	Cut point	Sensitivity	Specificity
Medial temporal composite (ERC+PHC+AMYG)	0.63	0.90	0.82	1.26	0.71	0.92
Temporal composite	0.58	0.90	0.81	1.20	0.64	0.93
Entorhinal	0.57	0.88	0.82	1.49	0.66	0.91
Parahippocampus	0.57	0.88	0.80	1.27	0.67	0.90
Inferior temporal	0.56	0.91	0.81	1.21	0.61	0.95
Amygdala	0.55	0.89	0.81	1.03	0.63	0.92
Fusiform	0.53	0.85	0.80	1.19	0.66	0.87
Middle temporal	0.51	0.85	0.77	1.15	0.63	0.88
Lateral parietal	0.47	0.87	0.75	1.10	0.57	0.90
Posterior cingulate	0.39	0.72	0.75	1.06	0.66	0.73
Hippocampus	0.38	0.78	0.71	1.20	0.58	0.80

eTable 2. AUROC results using predicted probabilities derived from Gaussian mixture models (GMM).
GMM were run using the “mclust” package in R.

CU Aβ- (n=599) vs MCI Aβ+ (n=48)

ROI SUVR	Youden	Accuracy	AUC	Sensitivity	Specificity
Medial temporal composite (ERC+PHC+AMYG)	0.61	0.89	0.87	0.71	0.90
Entorhinal	0.56	0.84	0.82	0.71	0.85
Temporal composite	0.56	0.87	0.82	0.67	0.89
Parahippocampus	0.55	0.87	0.84	0.67	0.88
Inferior temporal	0.54	0.89	0.78	0.62	0.91
Amygdala	0.50	0.94	0.78	0.52	0.97
Fusiform	0.48	0.76	0.78	0.71	0.77
Middle temporal	0.45	0.90	0.73	0.52	0.93
Lateral parietal	0.37	0.79	0.67	0.56	0.81
Hippocampus	0.34	0.71	0.68	0.62	0.71
Posterior cingulate	0.25	0.62	0.64	0.62	0.62

AUROC results using predicted probabilities derived from Gaussian mixture models. CU Aβ- (n=599) vs AD Aβ+ (n=28)

ROI SUVR	Youden	Accuracy	AUC	Sensitivity	Specificity
Temporal composite	0.66	0.97	0.85	0.68	0.98
Medial temporal composite (ERC+PHC+AMYG)	0.66	0.94	0.83	0.71	0.95
Inferior temporal	0.65	0.96	0.87	0.68	0.97
Entorhinal	0.63	0.94	0.86	0.68	0.95
Middle temporal	0.63	0.97	0.83	0.64	0.98
Parahippocampus	0.62	0.93	0.86	0.68	0.94
Amygdala	0.62	0.90	0.82	0.71	0.91
Fusiform	0.61	0.96	0.82	0.64	0.97
Lateral parietal	0.58	0.86	0.84	0.71	0.86
Posterior cingulate	0.57	0.91	0.80	0.64	0.93
Hippocampus	0.51	0.82	0.77	0.68	0.83

AUROC results using predicted probabilities derived from Gaussian mixture models. CU Aβ- (n=599) vs CI Aβ+ (n=76)

ROI SUVR	Youden	Accuracy	AUC	Sensitivity	Specificity
Medial temporal composite (ERC+PHC+AMYG)	0.62	0.88	0.85	0.72	0.90
Inferior temporal	0.57	0.90	0.81	0.64	0.93
Parahippocampus	0.57	0.86	0.84	0.68	0.88
Temporal composite	0.57	0.87	0.83	0.68	0.89
Entorhinal	0.56	0.88	0.84	0.66	0.90
Amygdala	0.52	0.93	0.80	0.55	0.97
Fusiform	0.49	0.76	0.80	0.72	0.77
Middle temporal	0.49	0.89	0.77	0.57	0.93
Lateral parietal	0.44	0.82	0.73	0.59	0.85
Hippocampus	0.39	0.79	0.71	0.57	0.82
Posterior cingulate	0.35	0.87	0.70	0.42	0.93

eTable 3. Amyloid & tau status by cognitive and ethnoracial group
Participants were classified as tau positive if their medial temporal ROI exceeded 1.26

	CU			*MCI*			*Dementia*		
	NHW (N=323)	Hispanic (N=193)	NHB (N=213)	NHW (N=39)	Hispanic (N=45)	NHB (N=36)	NHW (N=13)	Hispanic (N=8)	NHB (N=9)
A-T-	231 (71.5%)	157 (81.3%)	166 (77.9%)	18 (46.2%)	30 (66.6%)	17 (47.2%)	1 (7.7%)	1 (12.5%)	0 (0%)
A-T+	18 (5.6%)	10 (5.3%)	17 (8.0%)	2 (5.1%)	3 (6.7%)	2 (5.6%)	0 (0%)	0 (0%)	0 (0%)
A+T-	49 (15.2%)	19 (9.8%)	19 (8.9%)	5 (12.8%)	0 (0%)	11 (30.6%)	3 (23.1%)	3 (37.5%)	1 (11.1%)
A+T+	25 (7.7%)	7 (3.6%)	11 (5.2%)	14 (35.9%)	12 (26.7%)	6 (16.6%)	9 (69.2%)	4 (50.0%)	8 (88.9%)

Amyloid status by cognitive and ethnoracial group

	CU			*MCI*			*Dementia*		
	NHW (N=323)	Hispanic (N=193)	NHB (N=213)	NHW (N=39)	Hispanic (N=45)	NHB (N=36)	NHW (N=13)	Hispanic (N=8)	NHB (N=9)
Aβ status									
-	249 (77.1%)	167 (86.5%)	183 (85.9%)	20 (51.3%)	33 (73.3%)	19 (52.8%)	1 (7.7%)	1 (12.5%)	0 (0%)
+	74 (22.9%)	26 (13.5%)	30 (14.1%)	19 (48.7%)	12 (26.7%)	17 (47.2%)	12 (92.3%)	7 (87.5%)	9 (100%)

eTable 4. AUROC results using SUVRs in the full spectrum of participants, irrespective of amyloid status. In the full spectrum of participants regardless of amyloid status, AUROC analyses showed that continuous global Aβ best differentiated CU from dementia, with the highest Youden index (0.76). Discrimination for all measures was weaker for CU vs. MCI and CU vs. CI, with Youden indexes below 0.35, suggesting that tau cut points are effective in explaining cognitive status predominantly in amyloid positive individuals.

CU (n=729) vs MCI (n=120), not considering amyloid status

ROI SUVR	Youden	Accuracy	AUC	Cut point	Sensitivity	Specificity
Amygdala	0.26	0.77	0.63	1.01	0.43	0.83
Temporal composite	0.26	0.72	0.63	1.15	0.51	0.75
Medial temporal composite (ERC+PHC+AMYG)	0.26	0.76	0.63	1.23	0.44	0.81
Fusiform	0.24	0.70	0.64	1.16	0.50	0.74
Entorhinal	0.24	0.77	0.62	1.46	0.41	0.83
Parahippocampus	0.24	0.79	0.63	1.26	0.38	0.86
Global amyloid	0.23	0.74	0.63	1.07	0.44	0.79
Inferior temporal	0.23	0.75	0.63	1.15	0.42	0.81
Posterior cingulate	0.19	0.66	0.60	1.06	0.51	0.68
Lateral parietal	0.19	0.80	0.59	1.10	0.31	0.89
Middle temporal	0.17	0.69	0.60	1.11	0.44	0.73
Hippocampus	0.16	0.58	0.58	1.12	0.58	0.58

CU (n=729) vs. dementia (n=30), not considering amyloid status

ROI SUVR	Youden	Accuracy	AUC	Cut point	Sensitivity	Specificity
Global amyloid	0.76	0.83	0.90	1.08	0.93	0.83
Medial temporal composite (ERC+PHC+AMYG)	0.63	0.86	0.83	1.25	0.77	0.86
Temporal composite	0.61	0.96	0.84	1.27	0.63	0.98
Inferior temporal	0.60	0.95	0.84	1.26	0.63	0.96
Middle temporal	0.58	0.96	0.79	1.23	0.60	0.98
Amygdala	0.58	0.87	0.82	1.03	0.70	0.88
Fusiform	0.57	0.84	0.85	1.19	0.73	0.84
Entorhinal	0.57	0.86	0.83	1.49	0.70	0.87
Parahippocampus	0.57	0.81	0.82	1.23	0.77	0.81
Lateral parietal	0.56	0.79	0.80	1.06	0.77	0.79
Posterior cingulate	0.53	0.89	0.77	1.12	0.63	0.90
Hippocampus	0.49	0.81	0.79	1.22	0.67	0.82

CU (n=729) vs CI (n=150), not considering amyloid status

ROI SUVR	Youden	Accuracy	AUC	Cut point	Sensitivity	Specificity
Global amyloid	0.33	0.75	0.68	1.07	0.54	0.79
Amygdala	0.32	0.80	0.67	1.03	0.44	0.88
Medial temporal composite (ERC+PHC+AMYG)	0.32	0.76	0.67	1.23	0.51	0.81
Temporal composite	0.31	0.72	0.67	1.15	0.56	0.75
Entorhinal	0.30	0.77	0.66	1.46	0.47	0.83
Parahippocampus	0.30	0.75	0.67	1.23	0.50	0.80
Fusiform	0.29	0.72	0.68	1.17	0.54	0.75
Inferior temporal	0.29	0.75	0.67	1.15	0.48	0.81
Lateral parietal	0.26	0.80	0.63	1.10	0.37	0.89
Middle temporal	0.24	0.77	0.64	1.15	0.39	0.85
Posterior Cingulate	0.23	0.66	0.64	1.06	0.55	0.68
Hippocampus	0.21	0.68	0.62	1.17	0.49	0.72

eTable 5. Mean ± SD of SUVRs across ethnoracial and cognitive groups in 675 participants

	CU			MCI			AD		
ROI SUVR	NHW (N=249)	Hispanic (N=167)	NHB (N=183)	NHW (N=19)	Hispanic (N=12)	NHB (N=17)	NHW (N=12)	Hispanic (N=7)	NHB (N=9)
Temporal composite	1.10 ± 0.07	1.10 ± 0.07	1.09 ± 0.08	1.32 ± 0.23	1.38 ± 0.26*	1.14 ± 0.17*	1.56 ± 0.52*	1.57 ± 0.67*	1.78 ± 0.73*
Medial temporal composite	1.14 ± 0.10	1.13 ± 0.09	1.12 ± 0.10	1.44 ± 0.25	1.55 ± 0.25*	1.23 ± 0.27*	1.55 ± 0.41*	1.58 ± 0.55*	1.69 ± 0.46
Fusiform	1.12 ± 0.08	1.11 ± 0.08	1.10 ± 0.08	1.34 ± 0.28*	1.34 ± 0.17*	1.16 ± 0.23*	1.69 ± 0.64	1.58 ± 0.68*	1.74 ± 0.69*
Entorhinal	1.32 ± 0.17	1.31 ± 0.15	1.29 ± 0.13	1.69 ± 0.31*	1.85 ± 0.32*	1.44 ± 0.33*	1.78 ± 0.50	1.79 ± 0.55	1.95 ± 0.50
Inferior temporal	1.09 ± 0.07	1.10 ± 0.07	1.07 ± 0.08	1.32 ± 0.28	1.43 ± 0.39*	1.12 ± 0.16*	1.64 ± 0.65	1.62 ± 0.79*	1.96 ± 0.94*
Hippocampus	1.07 ± 0.09*	1.13 ± 0.11*	1.12 ± 0.14*	1.21 ± 0.17	1.26 ± 0.14	1.11 ± 0.18	1.28 ± 0.21	1.43 ± 0.39	1.39 ± 0.26
Parahippocampus	1.16 ± 0.09	1.15 ± 0.09	1.13 ± 0.10	1.37 ± 0.20	1.45 ± 0.18*	1.20 ± 0.22*	1.52 ± 0.40*	1.50 ± 0.51*	1.68 ± 0.45*
Amygdala	0.93 ± 0.08	0.91 ± 0.08	0.92 ± 0.10	1.26 ± 0.31*	1.31 ± 0.29*	1.04 ± 0.29*	1.34 ± 0.43	1.48 ± 0.62	1.46 ± 0.47*
Middle temporal	1.06 ± 0.07	1.07 ± 0.07	1.05 ± 0.08	1.27 ± 0.30	1.36 ± 0.38*	1.09 ± 0.13*	1.50 ± 0.57*	1.53 ± 0.72*	1.87 ± 0.96*
Posterior cingulate	1.03 ± 0.06	1.03 ± 0.07	1.02 ± 0.08	1.16 ± 0.21	1.17 ± 0.22	1.08 ± 0.10	1.26 ± 0.29*	1.41 ± 0.67	1.38 ± 0.33*
Lateral parietal	0.99 ± 0.07	1.02 ± 0.09*	0.98 ± 0.09*	1.12 ± 0.13	1.16 ± 0.23	1.04 ± 0.11	1.35 ± 0.41	1.46 ± 0.85	1.33 ± 0.37

* indicates significant post-hoc comparisons between ethnoracial group differences after FDR correction for multiple comparisons with α=0.05.

eFigure 5. SUVR Distributions by Diagnosis and Ethnoracial group

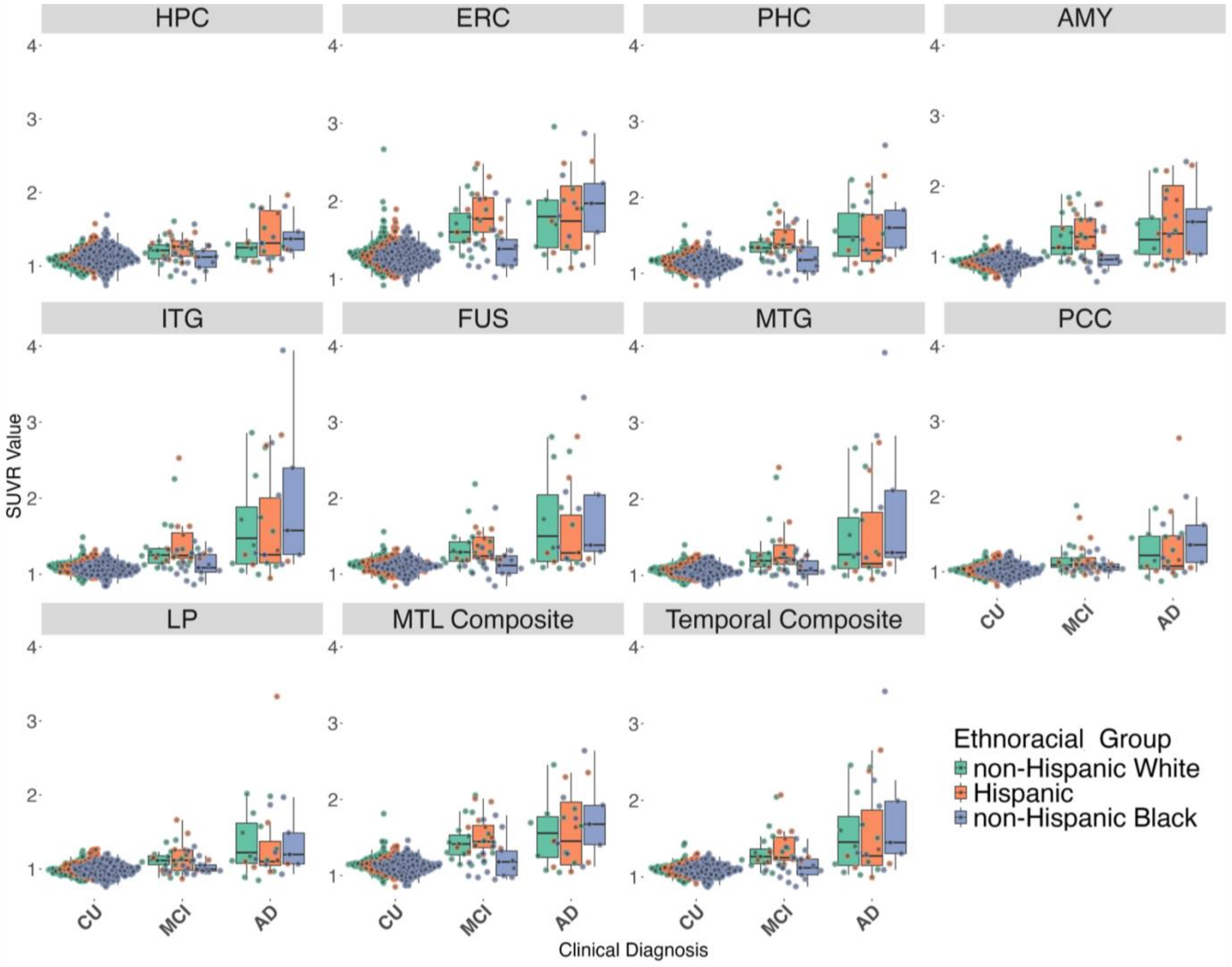

SUVR distributions by diagnosis and ethnoracial group in 675 participants.
HPC=Hippocampus, ERC=entorhinal cortex, PHC=parahippocampal cortex, AMY=amygdala, ITG=inferior temporal gyrus, FUS=fusiform gyrus, MTG=middle temporal gyrus, PCC=posterior cingulate cortex, LP=lateral parietal cortex, MTL=medial temporal lobe (ERC+PHC+AMY), temporal composite=HPC+ERC+PHC+AMY+ITG+FUS+MTG.

eTable 6. AUROC results fit in each ethnoracial group separately

Hispanic CU Aβ- (n=167) vs CI Aβ+ (n=19)

ROI SUVR	Youden	Accuracy	AUC	Cut point	Sensitivity	Specificity
Medial temporal composite (ERC+PHC+AMYG)	0.79	0.94	0.90	1.28	0.84	0.95
Entorhinal	0.76	0.91	0.91	1.50	0.84	0.92
Inferior temporal	0.73	0.84	0.90	1.17	0.89	0.83
Temporal composite	0.73	0.84	0.90	1.17	0.89	0.83
Fusiform	0.71	0.87	0.91	1.19	0.84	0.87
Amygdala	0.71	0.90	0.88	1.02	0.79	0.92
Parahippocampus	0.68	0.88	0.88	1.27	0.79	0.89
Middle temporal	0.61	0.85	0.84	1.15	0.74	0.87
Lateral parietal	0.51	0.85	0.72	1.10	0.63	0.88
Hippocampus	0.50	0.81	0.74	1.23	0.68	0.82
Posterior cingulate	0.39	0.74	0.73	1.07	0.63	0.75

NHB CU Aβ- (n=183) vs CI Aβ+ (n=26)

ROI SUVR	Youden	Accuracy	AUC	Cut point	Sensitivity	Specificity
Medial temporal composite (ERC+PHC+AMYG)	0.51	0.91	0.71	1.29	0.54	0.97
Inferior temporal	0.48	0.92	0.72	1.26	0.50	0.98
Middle temporal	0.47	0.88	0.70	1.18	0.54	0.93
Parahippocampus	0.47	0.91	0.71	1.31	0.50	0.97
Fusiform	0.46	0.87	0.70	1.21	0.54	0.92
Temporal composite	0.46	0.90	0.70	1.23	0.50	0.96
Entorhinal	0.45	0.75	0.72	1.37	0.69	0.76
Amygdala	0.38	0.92	0.68	1.30	0.38	1.00
Lateral parietal	0.35	0.75	0.71	1.05	0.58	0.78
Posterior cingulate	0.34	0.51	0.74	1.01	0.88	0.46
Hippocampus	0.24	0.86	0.60	1.31	0.31	0.93

NHW CU Aβ- (n=249) vs CI Aβ+ (n=31)

ROI SUVR	Youden	Accuracy	AUC	Cut point	Sensitivity	Specificity
Medial temporal composite (ERC+PHC+AMYG)	0.70	0.91	0.87	1.28	0.77	0.93
Amygdala	0.68	0.87	0.87	1.50	0.81	0.88
Parahippocampus	0.67	0.90	0.85	1.17	0.74	0.92
Temporal composite	0.65	0.84	0.85	1.17	0.81	0.84
Lateral parietal	0.63	0.89	0.80	1.19	0.71	0.92
Entorhinal	0.61	0.78	0.86	1.02	0.84	0.77

Inferior temporal	0.59	0.94	0.85	1.27	0.61	0.98
Fusiform	0.56	0.94	0.83	1.15	0.58	0.98
Middle temporal	0.55	0.85	0.81	1.10	0.68	0.88
Hippocampus	0.55	0.85	0.80	1.23	0.68	0.88
Posterior cingulate	0.51	0.76	0.76	1.07	0.74	0.77

eTable 7. Performance of the 1.26 cut-point in participants with high and low education

We also evaluated the performance of the 1.26 cut-point in subgroups defined by education level (received higher education vs. did not receive higher education, e.g., beyond high school). We assessed the sensitivity, specificity, and AUC of the cut-points in these subgroups. Delong's test indicated no statistical differences between AUCs (p= 0.23).

Group	N	Youden index	Sensitivity	Specificity	Accuracy	AUC
High Education	450	0.56	0.91	0.65	0.89	0.78
Low Education	205	0.69	0.95	0.74	0.92	0.85

Higher education = responses to the question: What educational or training degrees or certificates have you received since high school? {.00, none or part college} {1.00, vocational certificate} {2.00, associates} {3.00, bachelors} {4.00, masters} {5.00, doctoral}. Participants who had values greater than or equal to one were assigned 1 and those with zero were assigned 0. This variable was checked against the total number of years of education they received.

1. O'Bryant SE, Johnson LA, Barber RC, et al. The Health & Aging Brain among Latino Elders (HABLE) study methods and participant characteristics. *Alzheimers Dement (Amst)*. 2021;13(1):e12202. doi:10.1002/dad2.12202

2. Mormino EC, Toueg TN, Azevedo C, et al. Tau PET imaging with 18F-PI-2620 in aging and neurodegenerative diseases. *Eur J Nucl Med Mol Imaging*. 2021;48(7):2233-2244. doi:10.1007/s00259-020-04923-7

3. Song M, Beyer L, Kaiser L, et al. Binding characteristics of [18F]PI-2620 distinguish the clinically predicted tau isoform in different tauopathies by PET. *J Cereb Blood Flow Metab*. 2021;41(11):2957-2972. doi:10.1177/0271678X211018904

4. Lee N, Wheeler K, Tubi M, et al. Improved delineation of the reference region for 18F-PI-2620 tau PET analyses. Poster Presentation presented at: Society for Neuroscience; 2022; San Diego, CA.

5. Diedrichsen J, Balsters JH, Flavell J, Cussans E, Ramnani N. A probabilistic MR atlas of the human cerebellum. *Neuroimage*. 2009;46(1):39-46. doi:10.1016/j.neuroimage.2009.01.045

6. Diedrichsen J, Maderwald S, Küper M, et al. Imaging the deep cerebellar nuclei: a probabilistic atlas and normalization procedure. *Neuroimage*. 2011;54(3):1786-1794. doi:10.1016/j.neuroimage.2010.10.035

7. Landau S, Koeppe R, Jagust W. Florbetaben processing and positivity threshold derivation. Published online 2011.
